# Valproic Acid Causes Proteasomal Degradation of DICER and Influences miRNA Expression

**DOI:** 10.1371/journal.pone.0082895

**Published:** 2013-12-17

**Authors:** Zhaiyi Zhang, Paolo Convertini, Manli Shen, Xiu Xu, Frédéric Lemoine, Pierre de la Grange, Douglas A. Andres, Stefan Stamm

**Affiliations:** 1 Department of Molecular and Cellular Biochemistry, University of Kentucky, Lexington, Kentucky, United States of America; 2 GenoSplice Technology, Hôpital Saint-Louis, Av Claude Vellefaux, Paris, France; French National Center for Scientific Research - Institut de biologie moléculaire et cellulaire, France

## Abstract

Valproic acid (VPA) is a commonly used drug to treat epilepsy and bipolar disorders. Known properties of VPA are inhibitions of histone deacetylases and activation of extracellular signal regulated kinases (ERK), which cannot fully explain VPA’s clinical features. We found that VPA induces the proteasomal degradation of DICER, a key protein in the generation of micro RNAs. Unexpectedly, the concentration of several micro RNAs increases after VPA treatment, which is caused by the upregulation of their hosting genes prior to DICER degradation. The data suggest that a loss of DICER protein and changes in micro RNA concentration contributes to the clinical properties of VPA. VPA can be used experimentally to down regulate DICER protein levels, which likely reflects a natural regulation of DICER.

## Introduction

Valproic Acid (VPA) is a widely used drug to treat epilepsy [1], migraines [2] and bipolar disorders [3]. VPA is currently being tested for the treatment of additional diseases, such as spinal muscular atrophy, where it promotes inclusion of a critical alternative exon into the SMN2 pre-mRNA [4]. In addition, VPA is tested as an anti- cancer drug [5–7]. 

Despite its frequent clinical use, its mechanism of action is not fully understood. The drug has both acute (within days) and chronic (within two weeks) effects depending on the disease treated. VPA was shown to block histone deacetylase (HDAC) activity, suggesting one mode of action is changing gene expression *via* chromatin modifications [8]. In addition, it was found that VPA activates the ERK (extracellular signal-regulated kinases) pathway and subsequently influences AP-1-dependent gene expression [9]. However the molecular mechanisms remained elusive. In addition to its inhibition of enzymatic activities, VPA causes the proteasomal degradation of HADC2 [10] and CREM binding protein (CBP) [11].

The effect of VPA on gene expression has been tested in several cell systems using cDNA-based expression arrays. VPA treatment of rat cortical neurons generates 1,303 changes in mRNA expression [12]. Administration of VPA resulted in 121 changes in the brain of rats [13] and 11 changes in mice brains [14]. 

A change in gene expression does not only manifest itself in alterations of mRNA levels, but can also result in changes of non-coding RNAs, such as miRNAs (micro RNAs). miRNAs are 22 nt long RNAs generated from longer precursor RNAs. First, nuclear pri-miRNAs having characteristic secondary structures are cleaved by the drosha/DGCR8 complex in the nucleus to pre-miRNAs, which are transported into the cytosol where they are processed to mature miRNAs by the RNase III DICER. In general, miRNAs act in the repression of translation, but can also acquire other functions after binding to target RNAs [15]. Similar to other RNAs, the expression of some miRNAs is regulated by physiological stimuli, such as light/dark cycles in the retina [16]. DICER levels are regulated by autophagy through binding to the autophagy receptor NDP52 [17], which shows that miRNA production is under physiological regulation. Most miRNAs are relatively stable, with half-lives of several days in cell culture [18]. There are an increasing number of miRNAs shown to be involved in disease, for example, miR-134 is upregulated in epilepsy and its depletion reduces the occurrence of seizures [19].

Here, we analyzed the molecular mechanism of VPA, starting from genome-wide array analysis. Unexpectedly, we found that VPA causes the proteasomal degradation of DICER. In addition, VPA up regulates expression of several miRNA hosting genes, which results in an increase of a subset of miRNAs. Our data suggest that changes in miRNAs contribute to VPA’s clinical features.

## Results

### Genome-wide array analysis of HEK293 cells treated with Valproic Acid (VPA)

To investigate VPA’s molecular mechanism of action on gene expression, we performed Affymetrix exon junction array analysis using HEK293 cells. VPA is in clinical tests to treat spinal muscular atrophy [20], as it promotes inclusion of exon 7 of the SMN2 gene. Therefore, we used inclusion of the alternative exon 7 to determine the most effective concentration of VPA. Using a reporter gene in HEK293 cells, we found that 20 mM VPA gives the strongest effect after 12 hours of treatment (data not shown). 

**Figure 1:**
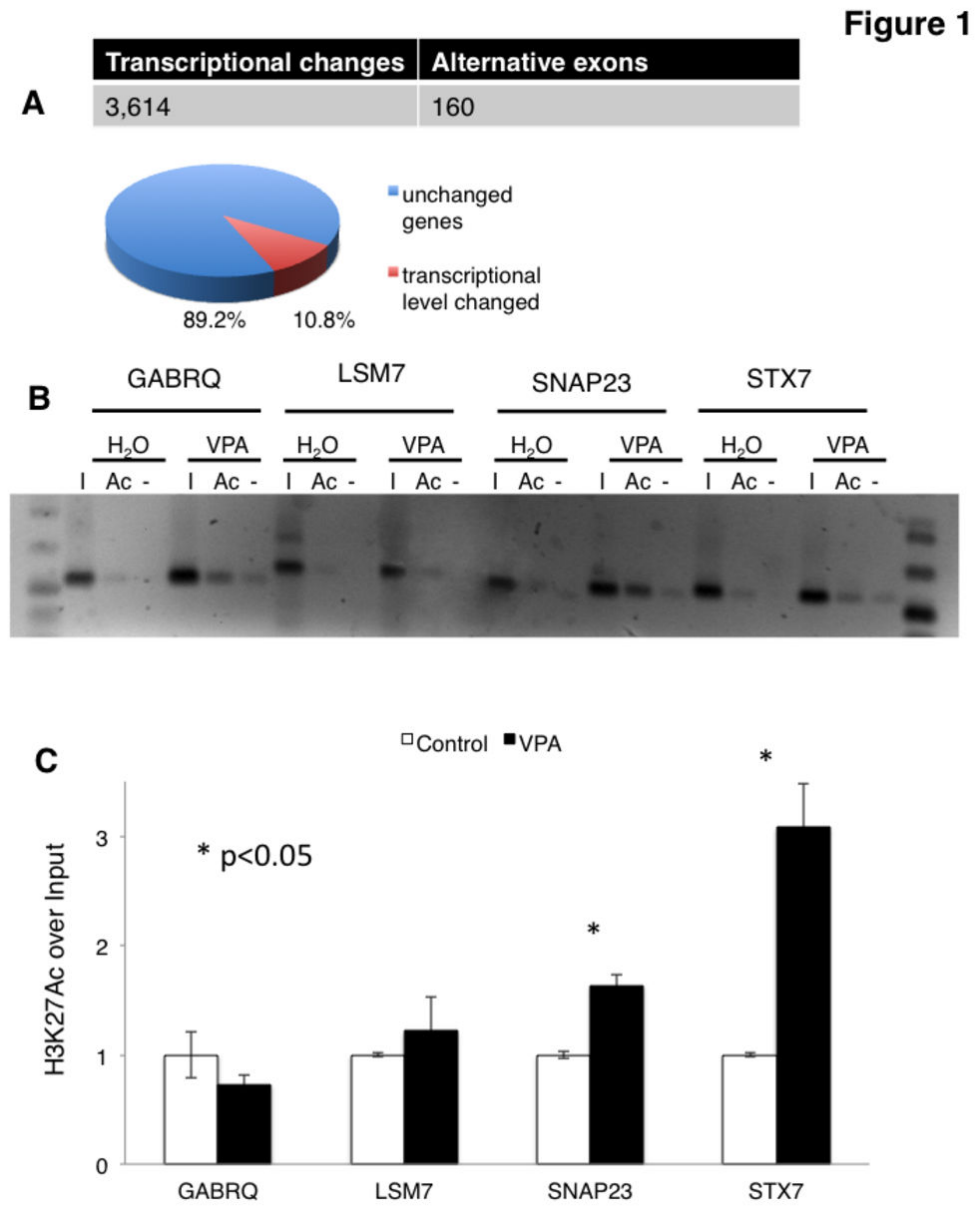
VPA changes gene expression. **A**. Overview of array analysis. HEK293 cells were treated for 16 hours with 20 mM Valproic Acid and compared with untreated cells using Affymetrix Exons Junction Arrays. About 11% of all human genes show changes on the transcriptional level, as indicated in the pie chart. **B**. Chromatin immunoprecipitations using an H3K27ac antiserum on HEK293 cell lysates after six hours of VPA treatment. Promoter regions of genes influenced by VPA (Figure S2) were tested. **C**. Statistical analysis of three independent experiments.

Thus, we tested the effect of 20 mM VPA in HEK293 cells after 16 hours of treatment using genome wide exon junction arrays. As shown in Figure 1A, VPA mainly changes the transcription levels but not the splice site selection. VPA changed 3,614 transcripts more than 1.5 fold, representing 10.8% of the 33,395 genes on the array. Unexpectedly, usage of only 160 alternative exons was changed. The most likely cause for the transcriptional deregulation is the inhibition of histone deacetylation, which we tested by chromatin immunoprecipitations. As expected, after six hours of VPA treatment, we see an increase in H3K27 acetylation in 3 out of 4 genes (Figure 1B, C), supporting the idea that an increase in histone acetylation causes most of the observed changes in gene expression.

 We next determined the biological pathways affected (Figure S1 ), defined by the KEGG database [21]. Unexpectedly for an HDAC inhibitor, we found statistically significant enrichment of genes affecting SNARE (Soluble NSF Attachment Protein Receptor) interactions in vesicular transport, MAPK pathways and endocytosis (p values < 10^-3^). This suggests that VPA treatment does not affect gene expression uniformly, but targets distinct pathways. The complete array analysis, including the changes in alternative splicing and the pathway analysis is shown in Figure S2 as a spreadsheet.

**Figure 2:**
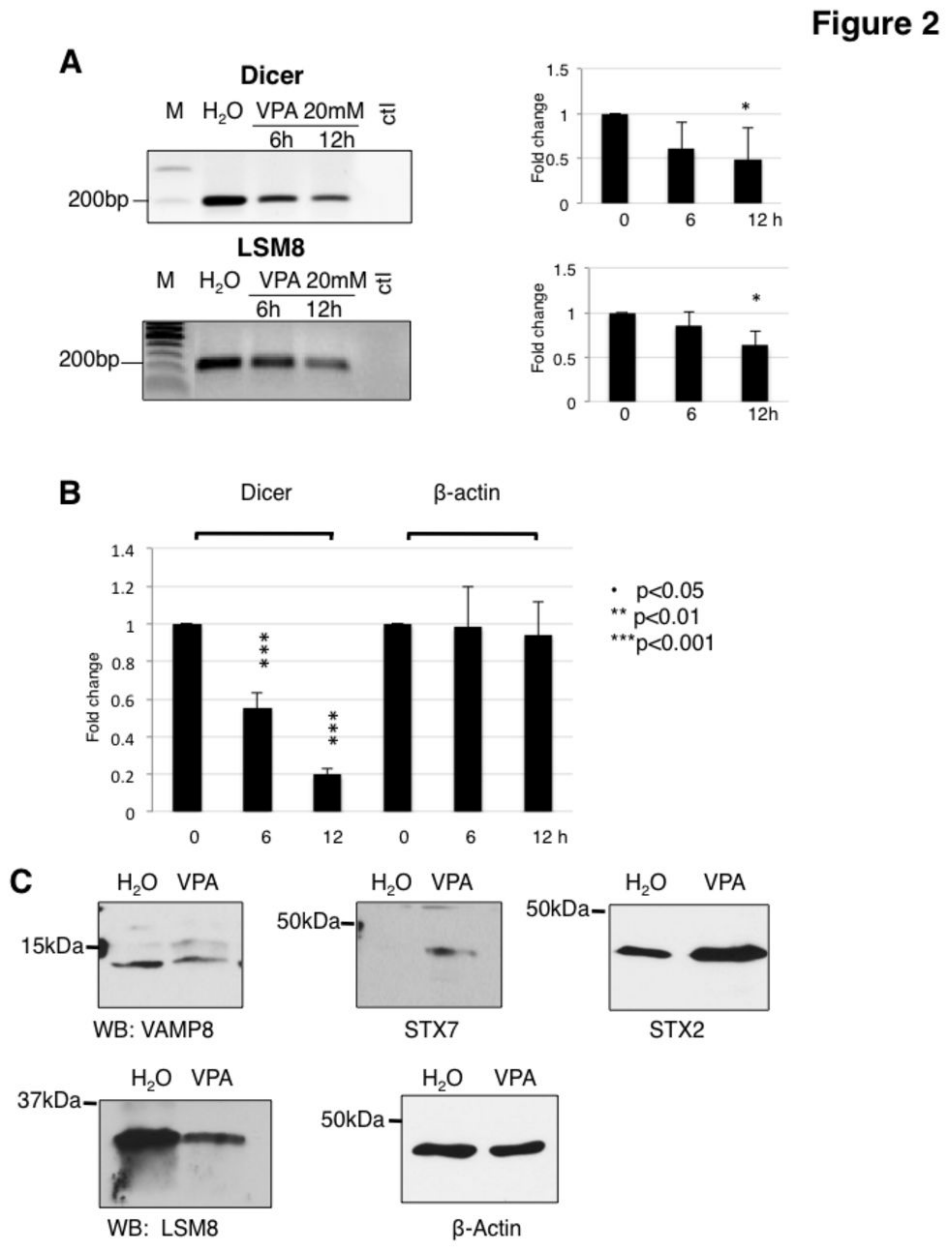
Validation of VPA induced changes. **A**. Effect of VPA on dicer and LSM8 mRNA expression after 6 and 12 hours of treatment, determined by RT-PCR (*: p < 0.05, from three independent experiments). H_2_O was used as negative control (ctl). **B**. Real time PCR analysis of DICER mRNA expression after VPA treatment for six and 12 hrs. **C**. Changes on protein level determined by Western Blot. HEK293 cells were treated for 16 hours with 20 mM VPA. Total cell lysates were separated by SDS page and analyzed using the antisera indicated. Beta-actin was used as a loading control. The mRNA changes of these genes are shown in Figure S3, VAMP8: downregulated, STX7: upregulated; STX2: downregulated; LSM8: downregulated.

RT-PCR analysis showed the array data to be highly reproducible with a validation rate of about 80% (13/16 genes tested, Figure S3; Figure 2A, B). All genes tested showed a change in gene expression that continuously increased from zero, six and 12 hours of VPA treatment. Due to its central role in miRNA production, we further validated the effect of VPA on dicer mRNA and by end-point and real time PCR, which supported the decrease of DICER mRNA indicated by the array analysis (Figure 2A, B).

In summary, the array analysis indicated that VPA mainly affects overall gene expression and causes a coordinated change in distinct biological pathways. The targeting of defined pathways is in agreement with VPA’s use as a drug causing specific physiological effects. 

### mRNA changes caused by VPA are reflected on the protein level

Next we used selected antisera to test whether the changes detected on mRNA levels are also apparent on protein levels. We performed Western Blot analysis on protein lysates from HEK293 cells treated for 16 hours with 20 mM VPA. As shown in Figure 1C, genes that change their expression levels after VPA treatment also alter the expression level of the encoded proteins.

In four of five cases, the change in protein expression correlated with the mRNA regulation, as a drop or increase in mRNA caused a similar change in protein concentration. However, the syntaxin 2 gene (STX2) showed a slight downregulation of mRNA (Figure S3), but a strong increase of protein level (Figure 2C). These data indicate that in addition to influencing gene expression, VPA has an additional, posttranscriptional effect on protein expression.

### VPA down regulates DICER protein levels

**Figure 3:**
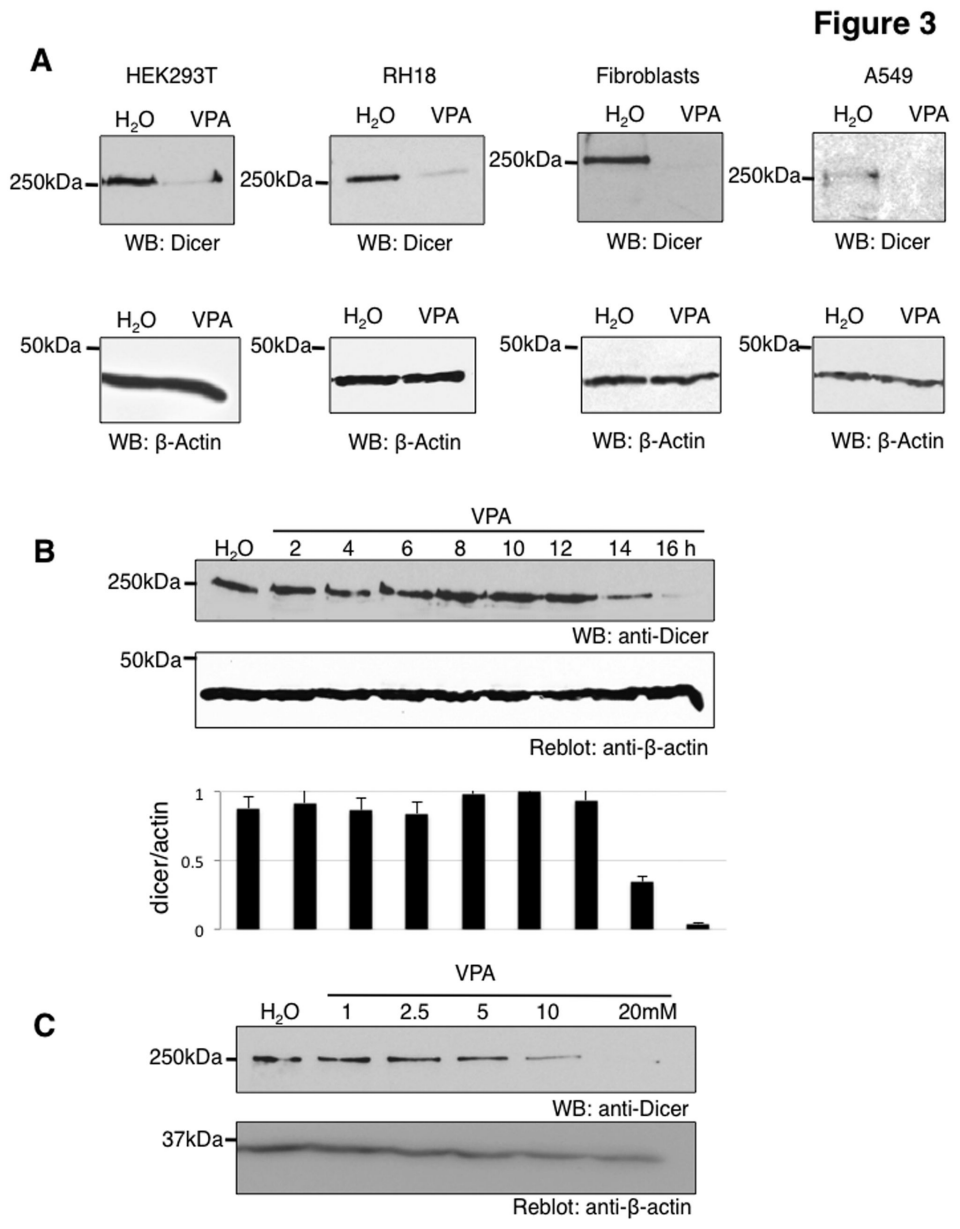
Influence of VPA on DICER protein expression levels. **A**. Effect of VPA on DICER protein level in different cell lines. The cell lines indicated were treated with 20 mM VPA for 16 hours and the expression of DICER was detected using Western blot. Beta actin was used as a loading control in each cell line. **B**. Time course of DICER expression in HEK293 cells treated with 20 mM VPA. DICER protein level is significantly reduced after 12 hours of VPA treatment. To quantify the effect, we calculated the intensity of DICER protein signal normalized to ß-actin, shown below, n=3. **C**. Concentration dependency of DICER degradation in HEK293 cells during 16 hours of treatment.

We next investigated DICER in more detail. Dicer mRNA is about 50% down regulated by VPA (Figure 2A). Using a previously described anti DICER antiserum [22], we detected a strong downregulation of DICER protein in HEK293 cells. We therefore raised a new DICER antiserum in goat (Figure S4) and found a similar down regulation of DICER protein (Figure 3A). After VPA treatment, DICER protein can only be detected by extended exposure of Western Blots made from HEK293 cells. A similar effect of DICER protein loss after VPA treatment was seen in Rhabdomyosarcoma cells (RH18) and adenocarcinomic human alveolar basal epithelial cells (A549), as well as in primary human fibroblasts (Figure 3A). We also tested HeLa cells and found that they express very low levels of DICER protein, which were reduced further by VPA treatment (data not shown). These data suggest that the reduction of DICER by VPA is common to cells.

### Time and concentration dependency of VPA acting on DICER protein levels

We next determined the concentration of VPA needed for DICER protein degradation and its time course. DICER protein levels significantly drop after 12 hours of treatment using 20 mM VPA (Figure 3B) in HEK293 cells, although mRNA can still be detected (Figure 2A). We then analyzed different concentrations at 16 hrs of treatment and found that VPA effectively reduces DICER protein when VPA is present in a concentration between five and 10 mM (Figure 3C). This concentration range is comparable to levels seen in serum of patients treated with VPA that range from 1-5 mM [23].

### VPA acts on DICER protein via the proteasome

**Figure 4:**
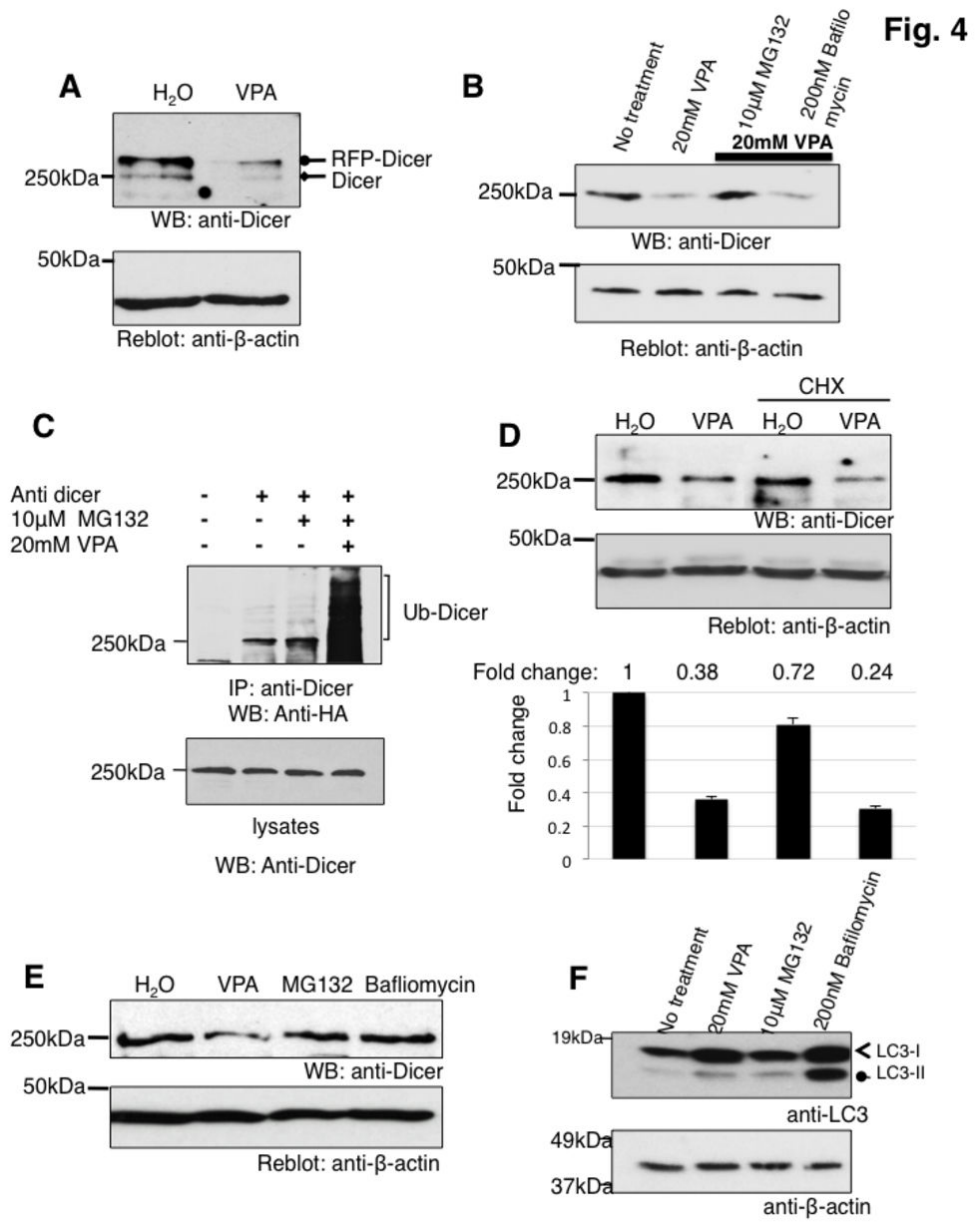
VPA leads to a degradation of the RNAse III DICER. **A**. Influence of VPA on DICER over expressed as a cDNA. HEK293 cells were transfected with RFP-tagged DICER and treated for 16 hours with 20 mM VPA. Proteins were separated on SDS page and analyzed by western blot using anti-DICER antisera. **B**. Proteasome inhibitors block the action of VPA on DICER. HEK293 cells were treated with 20 mM VPA in the presence of the proteasome inhibitor MG132 (10 µM), as well as the lysosome inhibitor bafliomycin A1 (200 nM) for 16 hours. Expression of endogenous DICER was detected using Western blot (top) and compared to ß-actin expression (bottom). The quantification is shown in Figure S5. **C**. Ubiquitination of DICER: HEK293 cells were transfected with a HA-tagged ubiquitin expression vector, in the presence of 10 µM MG132. Endogenous DICER was immunoprecipitated using anti-DICER antiserum, and analyzed with an anti-HA antiserum. VPA treatment was for 16 hours. The western blot with anti-DICER antiserum is shown below. **D**. Effect of VPA on cells after inhibition of translation. Cells were treated with cyclohexamide (100 mg/l) and 20 mM VPA for 12 hrs and DICER protein levels detected by western blot. ß-actin was used to demonstrate loading. The quantification underneath shows the fold change relative to the water control, set to 1, n=3. **E**. HEK293 cells were treated with 20 mM VPA, 10 µM MG132 and 200 nM bafliomycin A1 and DICER was detected by western blot. ß-actin was used as a loading control. The signal for DICER relative to actin was set to 1, VPA:0.36; MG132:0.93; Bafliomycin: 1. **F**. Response of LC3 protein levels to bafliomycin, MG132 and VPA treatment. Cells were treated for 12 hours with the drugs and the conversion of LC3-I to LC3-II was monitored by Western blot.

Next, we investigated whether VPA acts on the pre-mRNA of dicer and expressed DICER from cDNA constructs that lack introns. As shown in Figure 4A, VPA causes a strong decrease of both endogenous and RFP-tagged DICER, suggesting that VPA acts after pre-mRNA splicing.

Therefore, we tested the proteasome and lysosome as potential posttranslational degradation mechanism. Proteasomal degradation was blocked using the inhibitors MG132 [24]. Lysosomal action was inhibited using bafilomycin A1 [25]. As shown in Figure 4B, DICER degradation was blocked when the proteasome was inhibited by MG132, whereas the lysosomal inhibitor bafliomycin had no effect. The difference between MG132 and Bafliomycin on rescuing DICER degradation through VPA was significant (Figure S5), suggesting that VPA causes a proteasomal degradation.

To further analyze the role of the proteasome, we analyzed the ubiquitination of DICER protein and determined whether VPA causes a change in DICER ubiquitination. We immunoprecipitated endogenous DICER from cells transfected with HA-tagged ubiquitin expression constructs under denaturing conditions in RIPA buffer. In these experiments, the proteasome was blocked by addition of MG132. Probing these immunoprecipitates with an anti-HA antiserum detected a basal ubiquitination of DICER that is strongly increased when VPA is added to the cells (Figure 4C). Importantly, even in the absence of VPA, we can detect a small amount of ubiquitination of DICER, suggesting that ubiquitination and likely proteasomal degradation is part of a physiological regulation of DICER levels. 

We next blocked protein synthesis using cycloheximide and determined the effect of VPA on DICER expression. After 16 hrs of treatment, there is about 20% decrease of DICER protein, suggesting that DICER has a long half-life in HEK293 cells. The addition of VPA to cycloheximide-treated cells caused loss of DICER expression, suggesting that the effect is post-translational (Figure 4D).

As a further control, we treated cells with MB132 or Bafliomycin and observed no change in DICER expression, demonstrating that the loss of DICER is caused by VPA (Figure 4E). Since DICER was reported to be degraded by autophagy [17], we verified the effectiveness of bafliomycin using the microtubule-associated protein light chain 3 (LC3) as an autophagy marker [26]. As shown in Figure 4F, bafliomycin stabilized LC3-II, demonstrating that it blocks autophagy and worked in the assays. 

Together, the data suggest that similar to HDAC2 and CBP [10,11], VPA causes the proteasomal, but not lysosomal degradation of DICER.

### VPA Changes the miRNA Profile of HEK293 Cells

**Figure 5:**
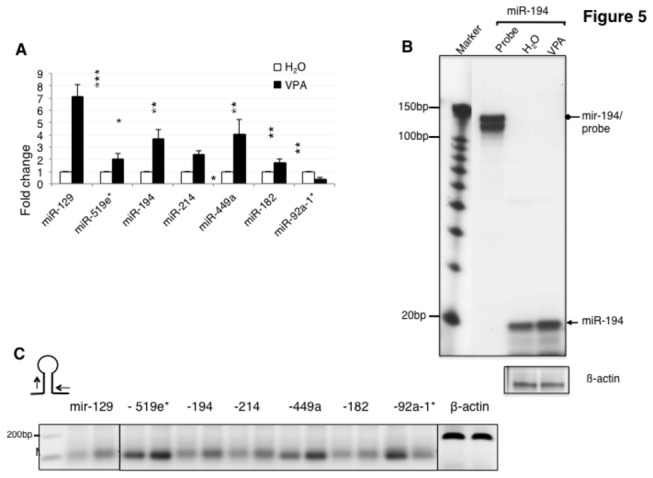
Change of miRNAs and their hosting genes after VPA treatment. **A**. Validation of miRNA expression changes detected by array analysis in VPA treated cells. Real-time PCR was performed using TaqMan probes (*: p < 0.05, **: p < 0.01, ***: p < 0.001, n>4). **B**. Validation of VPA-dependent miR-194 upregulation using RNAse protection. A probe complementary to pri-miR-194 was used and protected the mature miRNA. A precursor form is faintly visible (triangle). **C**. Changes in expression of the pri-miRNA genes detected by RT-PCR. The location of the miRNA amplification primers is schematically indicated. The primer sequences are given in Figure S8.

Human and mouse cells contain only one copy of the dicer gene critical to generate miRNAs. The homozygous disruption of dicer in mice is embryonic lethal, leads to the loss of miRNAs and an accumulation of double-stranded RNAs in stem cells [27]. However, several miRNAs can be made independently of DICER, for example by using the RNAse activity of argonaute 2 [28]. To test the effect of DICER depletion, we performed miRNA array analysis of HEK293 cells that were treated with 20 mM VPA for 16 hours. This treatment leads to an almost complete depletion of DICER protein (Figure 3A). From the 870 miRNAs present on the array, 117 human miRNAs were deregulated by VPA treatment. Unexpectedly, 49 miRNAs were markedly up regulated, despite the loss of DICER (Figure S6 and Figure S7). We next analyzed seven of the deregulated miRNAs by TaqMan analysis, which discriminates between mature miRNAs and pre-miRNAs. We could validate all changes in miRNAs tested (Figure 5A). Therefore, VPA up regulates some miRNAs, which is an unexpected property of the drug. The PCR-based assay was further validated for miR-194 using RNAse protection. We used a probe against the pri-miRNA and observed the upregulation of the mature miRNA (Figure 5B), which further validates the PCR results.

Since VPA upregulates several genes, it is possible that an increase of certain miRNAs is caused by an increase of their hosting genes. We therefore determined the concentration of pri-miRNAs hosting miRNAs that increase in response to VPA. We measured pri-miRNAs using RT-PCR employing flanking primers schematically indicated in Figure 5C. In cells treated for 16 hours with VPA we found that pri-miRNAs change similarly to the miRNAs they host. This suggests that VPA increases the expression of some miRNA hosting genes, which could explain the increase of some miRNAs after VPA treatment.

### VPA treatment changes translation from miRNA reporter constructs

**Figure 6:**
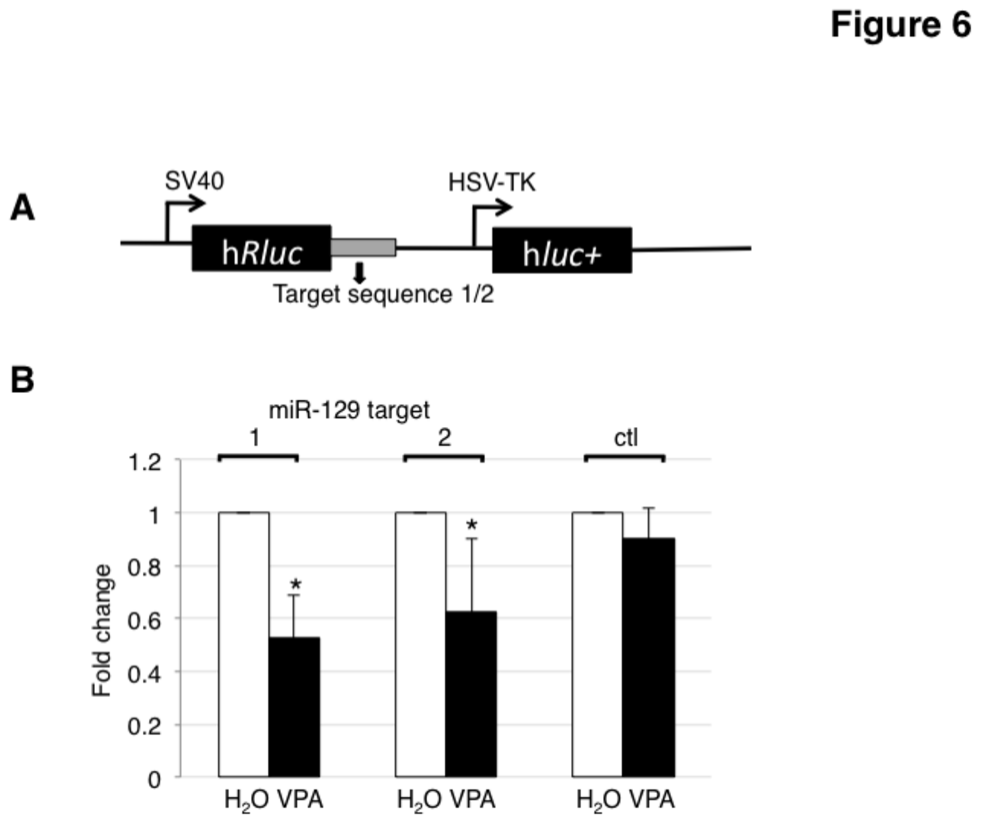
VPA regulates miR-129 containing reporter genes in cis and trans. **A**. Schematic diagram of the reporter construct indicating the location of the miRNA binding sites (small box) in the UTR of the hRluc gene. **B**. HEK293 cells were transfected with dual luciferase reporter genes containing miR-129 binding sites (1, 2). Renilla luciferase activity was subject to the regulation of miR-129 binding site. Firefly luciferase activity from the same reporter construct was used to normalize and to generate relative activity of Renilla luciferase. Ctl. is a luciferase construct without the miR-129 binding sites. The fold-change of relative luciferase activities Renilla/Firefly is shown. The transfected cells were treated with 20 mM VPA for 16 hours. The binding sequences for miR-129 were: 1: CACACAGCAAAAA; 2: GCAAAAA; Control sequence: TTTATTT.

Next, we determined whether VPA treatment could have a functional effect on miRNA targets. We used luciferase constructs containing miR-129 binding sites derived from the CDK6 3’ UTR as reporters, as this miRNA showed the strongest upregulation after VPA treatment and is a known regulator of CDK6 [29]. The reporters were transfected into HEK293 cells, which were subsequently treated with VPA for 16 hours. As shown in Figure 6A, B, we see a decrease in luciferase expression in constructs that contain the miR-129 binding site. Since miR-129 is highly up regulated by VPA (Figure 5A), this suggests that VPA-mediated changes in gene expression can influence translation of target genes

## Discussion

### VPA changes gene expression of distinct biological pathways

To gain insight into VPA’s molecular action, we performed genome-wide array analysis. We detected massive changes in overall gene expression that affected about 11% of the annotated genes. Most of the genes showed increased expression in response to VPA: 2,667 genes were up regulated and 947 were down regulated, which is in agreement with an inhibition of histone deacetylation that opens chromatin for transcription. Using H3K27Ac as an acetylation marker, we observed fast changes in three out of four genes. It is likely that VPA affects other histone acetylation events as well. VPA did not affect pathways indiscriminately, as pathways dealing with intracellular traffic and MAP kinases were disproportionally affected, indicating VPA has additional mechanisms of action.

### VPA causes a loss of DICER protein

Using array analysis and PCR-validation, we detected a modest down regulation of the dicer pre-mRNA. A subsequent validation of the effect on the protein level showed a loss of DICER protein. DICER is hardly detectable after 16 hours of VPA treatment in all cell lines tested. The DICER protein loss is also seen when the protein is generated from transfected cDNAs, suggesting a posttranslational mechanism. The proteasomal inhibitor MG132 blocks the disappearance of DICER. Using over expressed ubiquitin, we could detect ubiquitination of DICER that is strongly increased by VPA treatment, indicating VPA promotes an ubiquitination of DICER by an unknown mechanism. 

It has been recently reported that DICER is degraded by autophagy [17]. Since we cannot reverse the effect of VPA using the lysosomal inhibitor bafliomycin, but can reverse the effect with the proteasomal inhibitor MG132, it is likely that VPA causes degradation only in the proteasome. This suggests that cells can degrade DICER both in the proteasome and through autophagy using distinct pathways. 

To our knowledge, proteasomal degradation of DICER has not been reported. Since there is detectable ubiquitination of DICER in untreated HEK293 cells, it is possible that VPA enhances a naturally signaling pathway controlling DICER degradation. The ubiquitination of DICER is low in untreated cells, and physiological signals that increase the ubiquitination remain to be determined.

VPA promotes similar proteasomal degradation of two other proteins, HADC2 [10] and CREM binding protein (CBP) [11], suggesting targeted protein degradation is part of the VPA’s mode of action. It is possible VPA causes the degradation of other proteins as well. Previously, a genome-wide proteomics analysis revealed DICER is associated with the deubiquitination enzyme USP49 [30], which further supports endogenous ubiquitination of DICER. It is possible VPA interferes with USP49 acting on DICER. We also noticed that VPA causes a downregulation of LSM8/NAA38. This protein has a dual function as a spliceosomal U6 component and a component of the N-(alpha)-acetyltransferase complex that co-translationally transfers an acetyl group to the first methionine of certain proteins [31]. It is possible that loss of this acetylation contributes either directly of indirectly to the degradation of DICER. 

### VPA paradoxically increases the concentration of some miRNAs

The loss of DICER protein is expected to reduce miRNAs, as canonical miRNAs disappear in genetic dicer knockouts [27,32,33]. We tested this hypothesis using genome-wide miRNA arrays and surprisingly found that most miRNAs do not change significantly after DICER loss. This is likely due to the stability of miRNAs, which has been estimated to be around five days for most miRNAs [18]. We found some miRNAs to be reduced within 16 hours of VPA treatment, which reflects about four hours without DICER protein. This supports earlier findings that some miRNAs are relatively unstable and undergo rapid turnover [16,18].

VPA treatment causes almost complete degradation of DICER, but does not change expression of 87% of miRNAs. This suggests that VPA acts predominantly on DICER that is free from bound miRNAs.

Unexpectedly, we found VPA up regulates some miRNAs, which is a unique feature of VPA’s mode of action. The up regulation is likely due to the different time course for VPA mediated increase in transcription of miRNA hosting genes and the decrease of DICER protein through degradation. Transcriptional changes occur within six hours of VPA treatment, whereas it takes more than 12 hours for DICER protein to significantly drop in cell culture. This could explain why we found an increase of several miRNAs after VPA treatment, as the hosting genes of these miRNAs are activated by VPA, likely through an increase of histone acetylation in their promoters. It is however possible that the up regulated miRNAs are generated by a DICER independent mechanism, for example through argonaute2 that processes some miRNAs [28].

An analysis of pathways common to all up regulated miRNAs using DIANA [34] identified “melanogenesis” as the most affected pathway. Valproic acid has been shown to increase melanin production and inhibit the proliferation of melanoma cells [35]. It remains to be determined whether the effect of VPA on melanoma cells is acting through a change in miRNAs.

The influence of VPA on both transcriptional regulation and DICER expression distinguishes VPA action from genetic dicer knockouts or siRNA mediated changes that show a general loss of mature miRNAs [27,33,36]. 

Due to its short half-life of 9-12 hours in humans, VPA serum concentrations fluctuate between peak and trough levels in serum [37]. It could therefore be possible that part of VPA’s mode of action is the repeated increase of pri-miRNA expression and dicer reduction, which could result in an increase of some miRNAs. In addition, it is possible that DICER levels recover during trough times in patients, which could contribute to the physiological response, as it allows the generation of miRNAs from an altered pri-miRNA pool.

### Regulating DICER protein concentration is a physiological event

Dicer mRNA expression is controlled by extracellular signals, such as serum withdrawal which reduces dicer mRNA [38]. Cellular stress caused by reactive oxygen species and Ras activation reduces DICER protein levels [39], indicating DICER levels are regulated. On an organismal level, DICER protein is reduced by pathophysiological events in several diseases. DICER is decreased in geographic atrophy (GA), a form of age-related macular degeneration leading to blindness [40]. DICER is also reduced in dilated cardiomyopathy, which can be reversed by ventricle assist devices [33], demonstrating DICER deregulation can be reversed *in vivo*. 

In relation to the use of VPA as an anti-epileptic drug, the finding that DICER protein is reduced in temporal lobe epilepsy (TLE) with hippocampal sclerosis is most interesting [41]. Experimentally induced seizures in mice cause a similar loss of DICER in affected brain regions. VPA could interfere with these systems by upregulating miRNA hosting genes.

In summary, we found VPA is a useful drug in manipulating miRNA metabolism for research purposes. Regarding its medical use, we identified an unexpected property of Valproic Acid: it changes miRNA composition and uniquely upregulates some miRNAs by simultaneously changing the expression of miRNA hosting genes and by causing the degradation of DICER. It is likely this paradoxical increase of miRNAs contributes directly to the physiological effects of VPA. 

## Materials and Methods

### Exon junction arrays

RNA was isolated using Qiagen RNA purification columns. Its quality was determined by RNA integrity (RIN) number analysis and samples with a RIN >9.5 were used following the Affymetrix labeling procedure. 

For the analysis, the signal from Affymetrix human junction arrays (HJAY) was normalized using the “Probe scaling” method. The background was corrected with ProbeEffect from GeneBase [42]. The gene expression index was computed from probes were selected using ProbeSelect from GeneBase [42]. The gene expression signals were computed using these probes. Genes were considered expressed if the mean intensity was ≥ 500. Genes were considered regulated if (1) they were expressed in at least one condition (for example, VPA and/or control), (2) the fold-change was greater or equal than 1.5 and (3) the unpaired t-test p-value between gene intensities was ≤ 0.05. For each probe, a splicing-index was computed. Unpaired t-tests were performed to determine the difference in probe expression between the two samples as described previously [43]. Probe p-values in each probeset were then summarized using Fisher's method. Using annotation files, splicing patterns (cassette exons, 5’/3’ alternative splice sites and mutually exclusive exons) were tested for a difference between isoforms, selecting the ones with a minimum number of regulated probeset (with a p-value ≤ 0.01) in each competing isoform (at least one third of “exclusion” probesets have to be significant and at least one third of “inclusion” probesets have to be significant and show an opposite regulation for the splicing-index compared to the “exclusion” probesets). For example, for a single cassette exon, the exclusion junction and at least one of the three inclusion probesets (one exon probeset and two inclusion junction probesets) have to be significant and have to show an opposite regulation for the splicing-index. 

### Pathway Analysis

Significant KEGG pathways [21] were retrieved using DAVID [44]. The p-values given are essentially more stringent Fisher’s Exact Tests, as the classical Fisher’s p-value is calculated after subtracting 1 from the number of genes changed in a given pathway, which is then analyzed using Fisher’s Exact Test. 

### MicroRNA microarray

RNA was isolated using Trizol (Invitrogen). Its quality was determined by RNA integrity (RIN) number analysis and samples with a RIN >9.5 were used following the Affymetrix labeling procedure for miRNA Array version 1.0. Data were analyzed using Affymetrix Expression Console™ Software. 

### Cell culture

The HEK293, HeLa, and A549 cells were obtained from the American Type Culture Collection (ATCC). RH18 cells were obtained from St. Jude Children’s Research Hospital and were described in [45]. The primary human fibroblast cells were obtained from Coriell Institute (GM 00498D), described in [46]. All the cells were grown in the recommended medium containing 10% fetal bovine serum (FBS) at 37 °C under 5% CO_2_.

### Cloning

For the construction of pRFP-Dicer, the PCR-amplified full length coding Dicer sequence was inserted into pRFP vector (a generous gift from Dr. Emilia Galperin) using the restriction sites NheI and AgeI.

### Western Blot

Cells were washed with PBS and then lysed using 200 μl RIPA buffer on ice for 20 minutes. Lysates were collected in Eppendorf tubes, cleared by centrifugation for 1 minute at 12,000 rpm. The supernatant was collected and the concentration was measured using a Bradford staining (Bio-Rad). Equal amounts of protein from each sample were loaded on SDS polyacrylamide gels. Proteins resolved on SDS polyacrylamide gels were transferred to nitrocellulose membranes (Schleicher and Schuell) in transfer buffer for 45 minutes at 120 V. After the transfer, the membranes were blocked three times for 20 minutes in 1 × NET-gelatine buffer at room temperature. Primary antibodies were diluted to 1:1000, then added and incubated overnight at 4 °C. The next day, the membranes were washed three times for 20 minutes in 1 × NET-gelatine and then incubated with horseradish peroxidase coupled secondary antibodies for 2 hours. The membranes were subsequently washed three times for 20 minutes in 1 × NET-gelatine and the bound antibodies were detected using the ECL system. 

### RT-PCR

400 ng of total RNA (200 ng/µl), 5 pmol of reverse primer and 40 U of SuperScript III reverse transcriptase (Invitrogen) were mixed in 5 µl of RT buffer. To reverse transcribe the RNA, the reaction was incubated at 55 °C for 50 minutes. One-third of the RT reaction was used for cDNA amplification. The reaction was performed in 25 µl and contained 10 pmol of specific forward and reverse primers, 200 µM dNTPs, 1 x Taq polymerase buffer and 1 U of Taq DNA polymerase. The amplification was carried out in an Eppendorf PCR System Thermocycler under the following conditions: initial denaturation for 4 minutes at 94 °C, 30 cycles for 30 seconds at 94 °C, 30 seconds at 60 °C and an extension of 1 minute at 72 °C. After the last cycle, the reaction was held for 5 minutes at the extension temperature to complete the amplification of all products. 

The fold change was estimated as follows: 2^Ct(reference)-Ct(sample)^, where Ct (reference) and Ct (sample) were control group and VPA treatment group, respectively. For each experiment, at least three independent experiments were analyzed. 

### Drug treatment

Exponentially growing HEK293 cells were split at a density of about 3 x 10^5^ cells / 8 cm^2^. 24 hours later, cells were treated with sodium valproate (Sigma) dissolved in water at appropriate concentration for the indicated times in the different experiments. The final concentrations were, Bafilomycin A1 200 nM; MG132 10 µM, all from Sigma.

### TaqMan analysis

5 ng of total RNA, 1 x RT primer, 15 mM dNTPs, 50U of SuperScript III Reverse Transcriptase, 1 x Reverse Transcription buffer, 4U of RNase inhibitor were mixed in a 15 μl RT reaction. The reverse transcription was performed under the following condition: 30 minutes at 16 °C, 30 minutes at 42 °C and 5 minutes at 85 °C. 

One-fifth of the RT reaction was used for the qPCR reaction. The reaction was performed in 20 µl and contained 1 x TaqMan MicroRNA assay primer, 1 x TaqMan universal PCR Master Mix no AmpErase UNG (Applied Biosystems). The amplification was carried out in a Stratagene Mx3000p Thermocycler under the following conditions: initial denaturation for 10 minutes at 95 °C, 40 cycles for 15 seconds at 95 °C and an extension of 1 minute at 60 °C. The TaqMan assay number for miR-129 is 000590; for miR-519e* is 1166; for miR-194 is 000493; for miR-214 is 002306; for miR-449a is 001030; for miR-182 is 002334; for miR-92a-1* is 002137. A list of primers for validations is given in Figure S8.

### Antiserum generation

An anti-dicer serum was raised in goat using the peptide ETSVPGRPGSTKRRQC. The second and third bleeds were pooled and affinity purified using the peptide. The Anti-CD63 antibody was purchased from Abcam, and anti-β-actin antibody was obtained from Sigma.

### Luciferase assay

HEK293 cells were seeded in 24 well plates, and were transfected with psiCHECK2 dual luciferase reporter plasmids containing miR-129 targeting sites (1 µg/300,000 cells). 24 hours post transfection, cells were lysed and luciferase activities were measured according to manufacturer’s instruction using the Dual-Glo^TM^ Assay System (Promega). Data were normalized to the activity of the firefly luciferase expressed from the same psiCHECK2 vector as the internal control.

Sequence for miR-129 target 1: CACACAGCAAAAA

Sequence for miR-129 target 2: GCAAAAA

Sequence for control: TTTATTT

### In Vivo Ubiquitination Assay

HEK293 cells were seeded in 6 well plates. Cells were transfected with an HA-ubiquitin expressing plasmid and were subsequently treated with 20 mM VPA and 10 μM MG132 for 16 hours. Cells were lysed in RIPA buffer. HA-ubiquitin-conjugated proteins were purified with anti-Dicer antisera and protein A/G beads (Thermo Scientific). The presence of HA-ubiquitin–conjugated Dicer protein in the immunoprecipitates was detected by immunoblotting with the anti-HA monoclonal antibody 2-2.2.14 (Thermo Scientific).

### RNAse protection experiments

Were performed as previously described [47,48] using a probe against pri-miR-194 (hg19: chr11:64,658,827-64,658,911) and 40 µg of HEK293 total RNA and 80,000 cpm of ^32^P-labeled probe, generated with alpha UTP (800 Ci/mmol) as the only source of UTP.

### Chromatin immunoprecipitations

HEK293 cells, treated and not with VPA, were pelleted at 1,000 rpm at 4 °C and washed in ice-cold PBS buffer. The cell pellet was resuspended in ice-cold NP-40 lysis buffer (10 mM Tris [pH 7.4], 10 mM NaCl, 3 mM MgCl_2_, 0.5% NP-40, 0.15 mM spermine, 0.5 mM spermidine) and incubated on ice for 5 minutes. The solution was centrifuged at 3,000 rpm for 10 minutes. The nuclear fraction (pellet) was then resuspended in 1 ml of ice-cold MNase digestion buffer (10 mM Tris [pH 7.4], 15 mM NaCl, 60 mM KCl, 0.15 mM spermine, 0.5 mM spermidine, 1 mM CaCl_2_) and digested with 50 U/ml MNase at 37˙C for 12 minutes. The reaction was stopped by addition of EDTA and NP-40 to an end concentration of 0.01 M and 0.1% respectively. Reactions were digested with RNaseA (0.1 mg) for 1 hr at 42 °C and further treated with Proteinase K at 37˙C for 1 hour. The chromatin was immunoprecipitated for 14–16 h at 4°C using speciﬁc antibodies to H3K27ac (AbCam). After the incubation, chromatin immunoprecipitates were puriﬁed, and then 2 µl of each sample were analyzed by real-time PCR. Primers used were: GABRQF: cctcgctgctctctccttag; GABRQR: cgcgaaaaggaacagaggt; LSM7F: cgcctaaggggaaactgag; LSM7R:atcctccggttctgcttctc; SNAP23F: ttcctctacactcggggatg; SNAP23R: gctctggggaacagcttatg: STX7F: aggctggcttcaccagataa; STX7: Rttgcaagggagctgttcttt.

## Supporting Information

Figure S1 **Summary of the Exon-junction array analysis.** Kyoto Encyclopedia Genes and Genomes (KEGG) pathway analysis of the genes showing transcriptional changes. The p-value indicates the confidence that the given pathway is changed, calculated with a more stringent version of Fisher’s exact test.
(PPTX)

Figure S2 **Array analysis in Excel format.** Excel file summarizing the array analysis.
(XLS)

Figure S3 **Validation of Array Results by RT-PCR.** **A**. RT-PCR analysis of HEK293 cells that were treated for 0, 6 and 12 hours with 20 mM VPA.
**B**. Statistical evaluation of the changes in three independent experiments. The expression level in untreated HEK293 cells was set to 1. (*: p < 0.05, **: p < 0.01, ***: p < 0.001, n>4).
(PPTX)

Figure S4 **Testing of the DICER antisera.** Goats were immunized with the peptide ETSVPGRPGSTKRRQC and final sera were analyzed at the indicated dilutions. Similar to other DICER antisera, a cross reactivity is seen around 60kDa.
(PPTX)

Figure S5 **Quantification of the effect of MG132 and Bafliomycin on VPA mediated DICER degradation.** The DICER signal was normalized to beta-actin and the ratio in the water treated control was set to 1; n=3. A representative experiment is shown in Figure 4B.
(PPTX)

Figure S6 **Summary of miRNAs changes after VPA treatment.** HEK293 cells were treated with 20 mM VPA and changes in miRNAs monitored by array analysis. The table lists miRNAs with the highest fold-changes and their experimentally validated target genes from the literatures.
(PPTX)

Figure S7 **Array analysis in Excel format.** Summary of miRNAs changes after VPA treatment.
(XLSX)

Figure S8 **Primers used in the Exon-junction array validation by RT-PCR.**
(PPTX)
