# Supplementary material for: Valproic Acid Causes Proteasomal Degradation of DICER and Influences miRNA Expression
Source: PLoS One. 2013 Dec 17;8(12):e82895. doi: 10.1371/journal.pone.0082895 (PMC3866160; doi:10.1371/journal.pone.0082895)
Supplement: Figure S1 — Summary of the Exon-junction array analysis. Kyoto Encyclopedia Genes and Genomes (KEGG) pathway analysis of the genes showing transcriptional changes. The p-value indicates the confidence that the given pathway is changed, calculated with a more stringent version of Fisher’s exact test. (PPTX) [file pone.0082895.s001.pptx]

## Slide 1
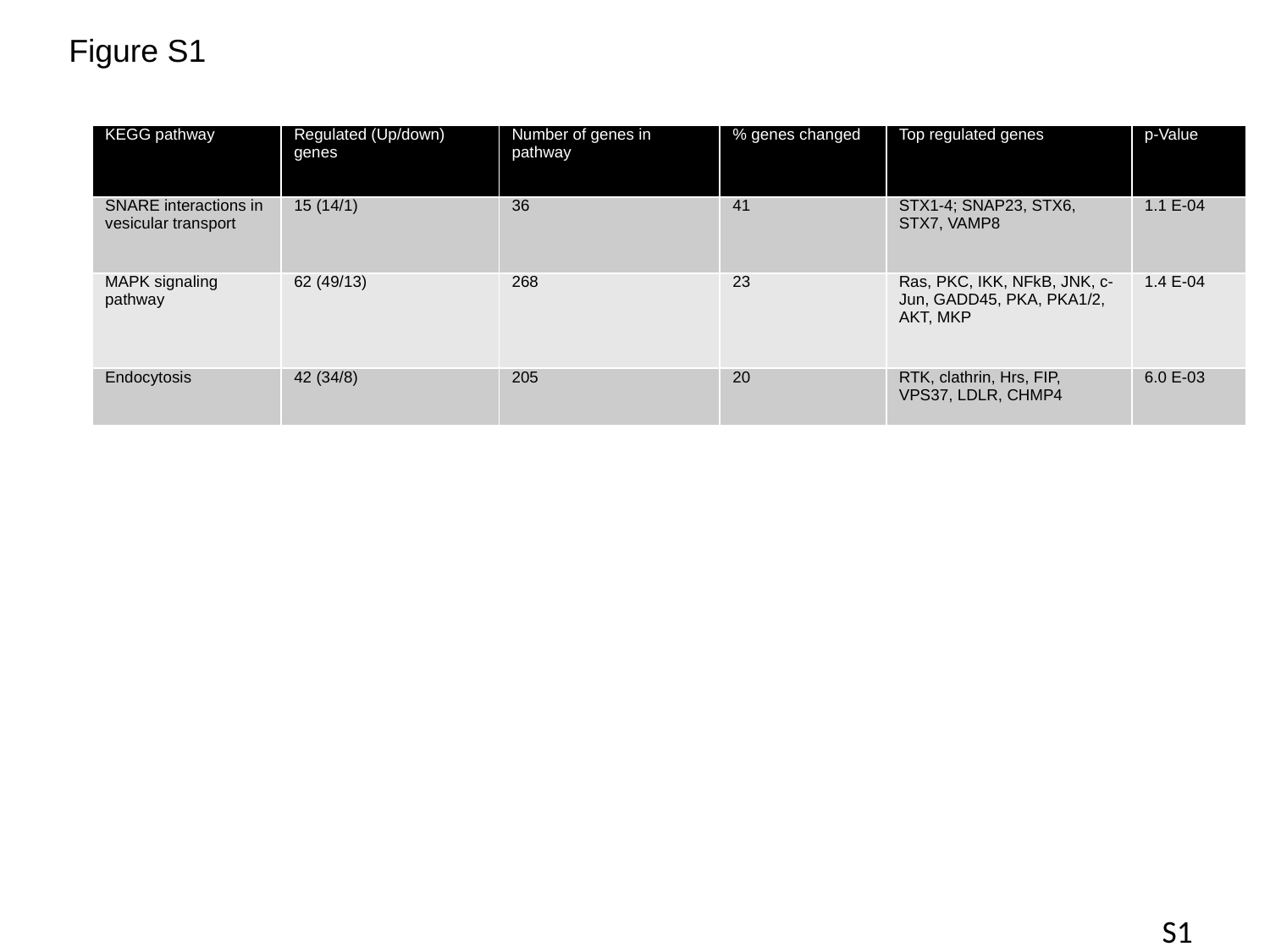

Figure S1
| KEGG pathway | Regulated (Up/down) genes | Number of genes in pathway | % genes changed | Top regulated genes | p-Value |
| --- | --- | --- | --- | --- | --- |
| SNARE interactions in vesicular transport | 15 (14/1) | 36 | 41 | STX1-4; SNAP23, STX6, STX7, VAMP8 | 1.1 E-04 |
| MAPK signaling pathway | 62 (49/13) | 268 | 23 | Ras, PKC, IKK, NFkB, JNK, c-Jun, GADD45, PKA, PKA1/2, AKT, MKP | 1.4 E-04 |
| Endocytosis | 42 (34/8) | 205 | 20 | RTK, clathrin, Hrs, FIP, VPS37, LDLR, CHMP4 | 6.0 E-03 |
S1
