# Supplementary material for: Valproic Acid Causes Proteasomal Degradation of DICER and Influences miRNA Expression
Source: PLoS One. 2013 Dec 17;8(12):e82895. doi: 10.1371/journal.pone.0082895 (PMC3866160; doi:10.1371/journal.pone.0082895)
Supplement: Figure S3 — Validation of Array Results by RT-PCR. A. RT-PCR analysis of HEK293 cells that were treated for 0, 6 and 12 hours with 20 mM VPA. B. Statistical evaluation of the changes in three independent experiments. The expression level in untreated HEK293 cells was set to 1. (*: p < 0.05, **: p < 0.01, ***: p < 0.001, n>4). (PPTX) [file pone.0082895.s003.pptx]

## Slide 1
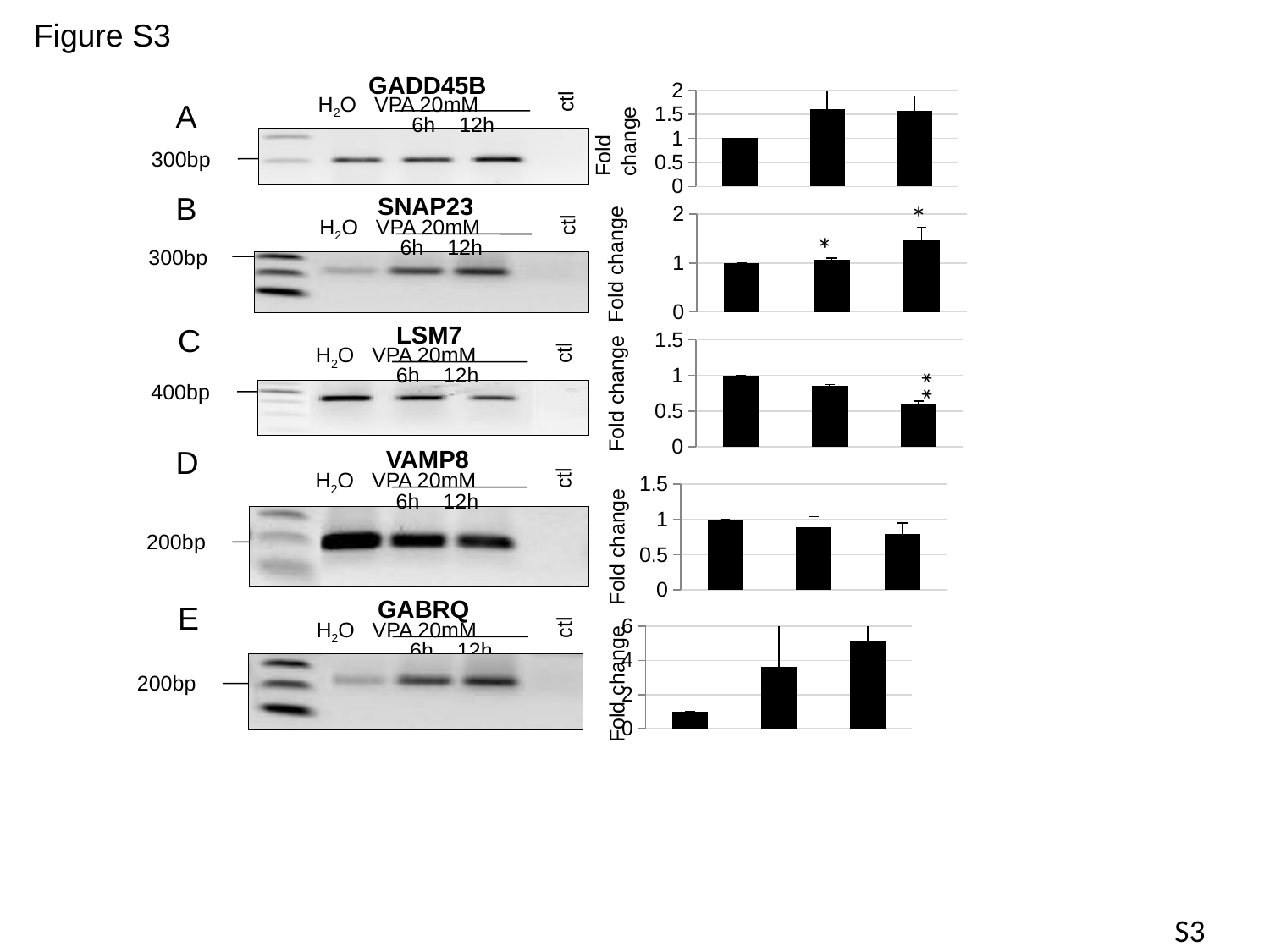

Figure S3
GADD45B
### Chart
| Category | |
|---|---|
| H2O | 1.0 |
| VPA6h | 1.60299530455373 |
| VPA12h | 1.566045022282421 |ctl
H2O VPA 20mM
A
Fold change
*
6h 12h
300bp
B
SNAP23
*
### Chart
| Category | |
|---|---|
| H2O | 1.0 |
| VPA6h | 1.068992739060695 |
| VPA12h | 1.467105019107536 |ctl
H2O VPA 20mM
*
6h 12h
300bp
Fold change
LSM7
C
### Chart
| Category | |
|---|---|
| H2O | 1.0 |
| VPA6h | 0.851277807502195 |
| VPA12h | 0.606737019075404 |ctl
H2O VPA 20mM
6h 12h
**
400bp
Fold change
D
VAMP8
ctl
H2O VPA 20mM
### Chart
| Category | |
|---|---|6h 12h
200bp
Fold change
GABRQ
E
ctl
H2O VPA 20mM
### Chart
| Category | |
|---|---|
| H2O | 1.0 |
| VPA6h | 3.630775117224114 |
| VPA12h | 5.140947343782343 |6h 12h
200bp
Fold change
S3

## Slide 2
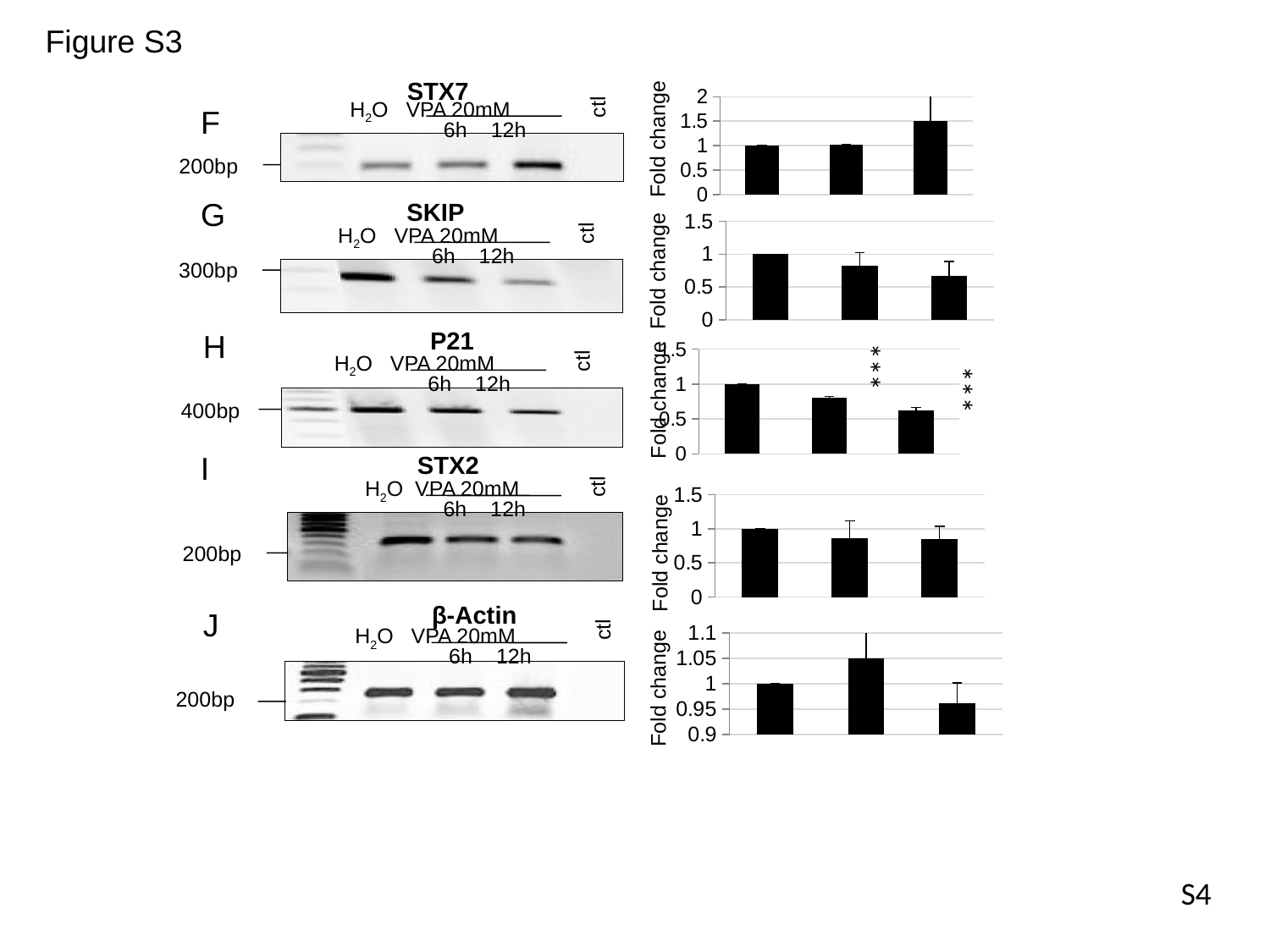

Figure S3
STX7
### Chart
| Category | |
|---|---|
| H2O | 1.0 |
| VPA6h | 1.01317144239913 |
| VPA12h | 1.509647023457988 |ctl
H2O VPA 20mM
F
6h 12h
Fold change
200bp
G
SKIP
### Chart
| Category | |
|---|---|
| H2O | 1.0 |
| VPA6h | 0.821253609189815 |
| VPA12h | 0.664653483836007 |ctl
H2O VPA 20mM
6h 12h
300bp
Fold change
P21
H
### Chart
| Category | |
|---|---|
| H2O | 1.0 |
| VPA6h | 0.803715784044256 |
| VPA12h | 0.628039398471202 |***
ctl
H2O VPA 20mM
***
6h 12h
Fold change
400bp
I
STX2
ctl
H2O VPA 20mM
### Chart
| Category | |
|---|---|6h 12h
200bp
Fold change
β-Actin
J
ctl
H2O VPA 20mM
### Chart
| Category | |
|---|---|
| H2O | 1.0 |
| VPA6h | 1.049219 |
| VPA12h | 0.960859 |6h 12h
Fold change
200bp
S4
