# Supplementary figures and images for: Valproic Acid Causes Proteasomal Degradation of DICER and Influences miRNA Expression
Source: PLoS One. 2013 Dec 17;8(12):e82895. doi: 10.1371/journal.pone.0082895 (PMC3866160; doi:10.1371/journal.pone.0082895)

## Slide 1
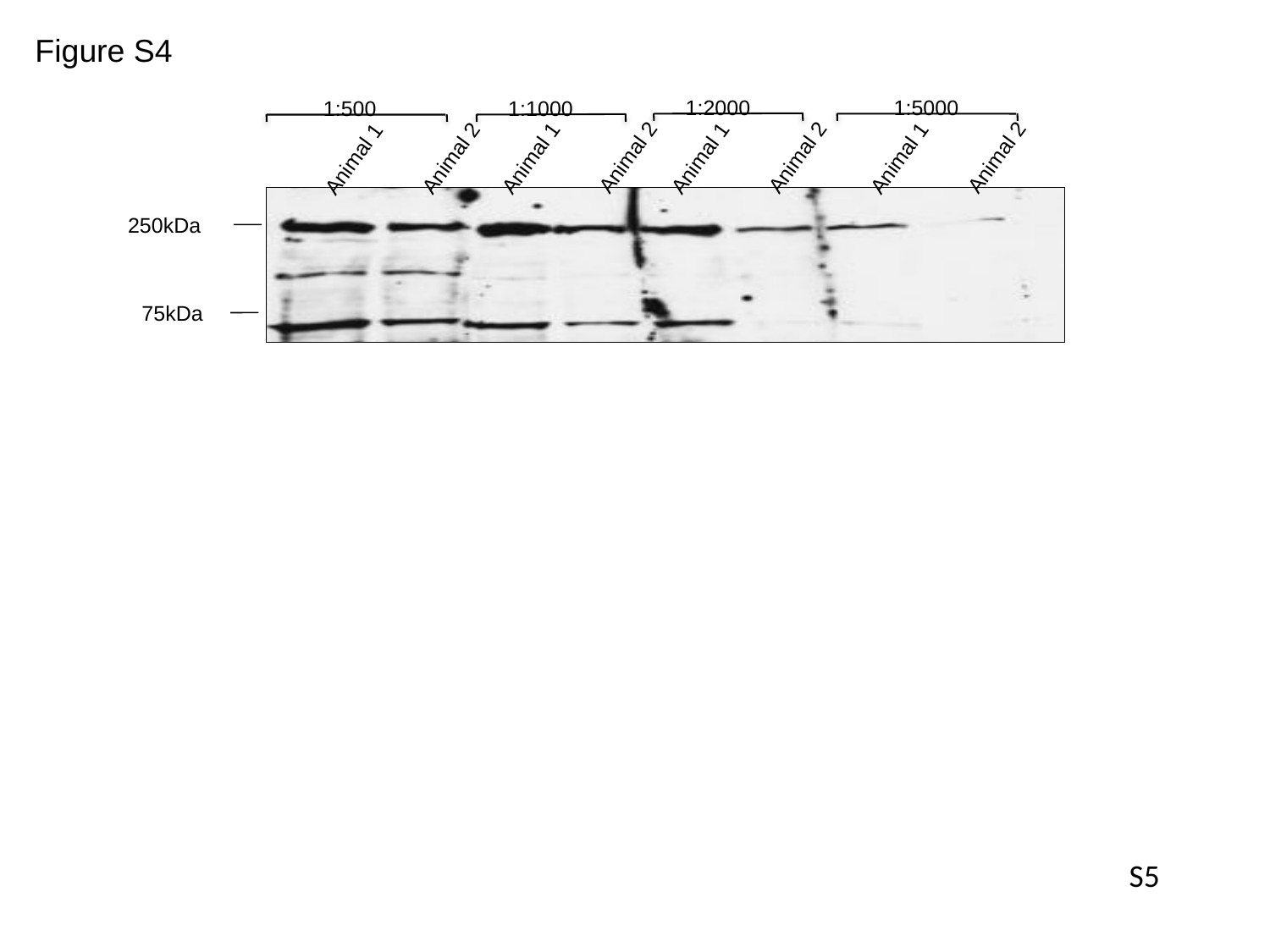

Figure S4
1:2000
1:5000
1:1000
1:500
Animal 2
Animal 2
Animal 2
Animal 1
Animal 1
Animal 2
Animal 1
Animal 1
250kDa
75kDa
S5

Supplement: Figure S4 — Testing of the DICER antisera. Goats were immunized with the peptide ETSVPGRPGSTKRRQC and final sera were analyzed at the indicated dilutions. Similar to other DICER antisera, a cross reactivity is seen around 60kDa. (PPTX) [file pone.0082895.s004.pptx]

## Slide 1
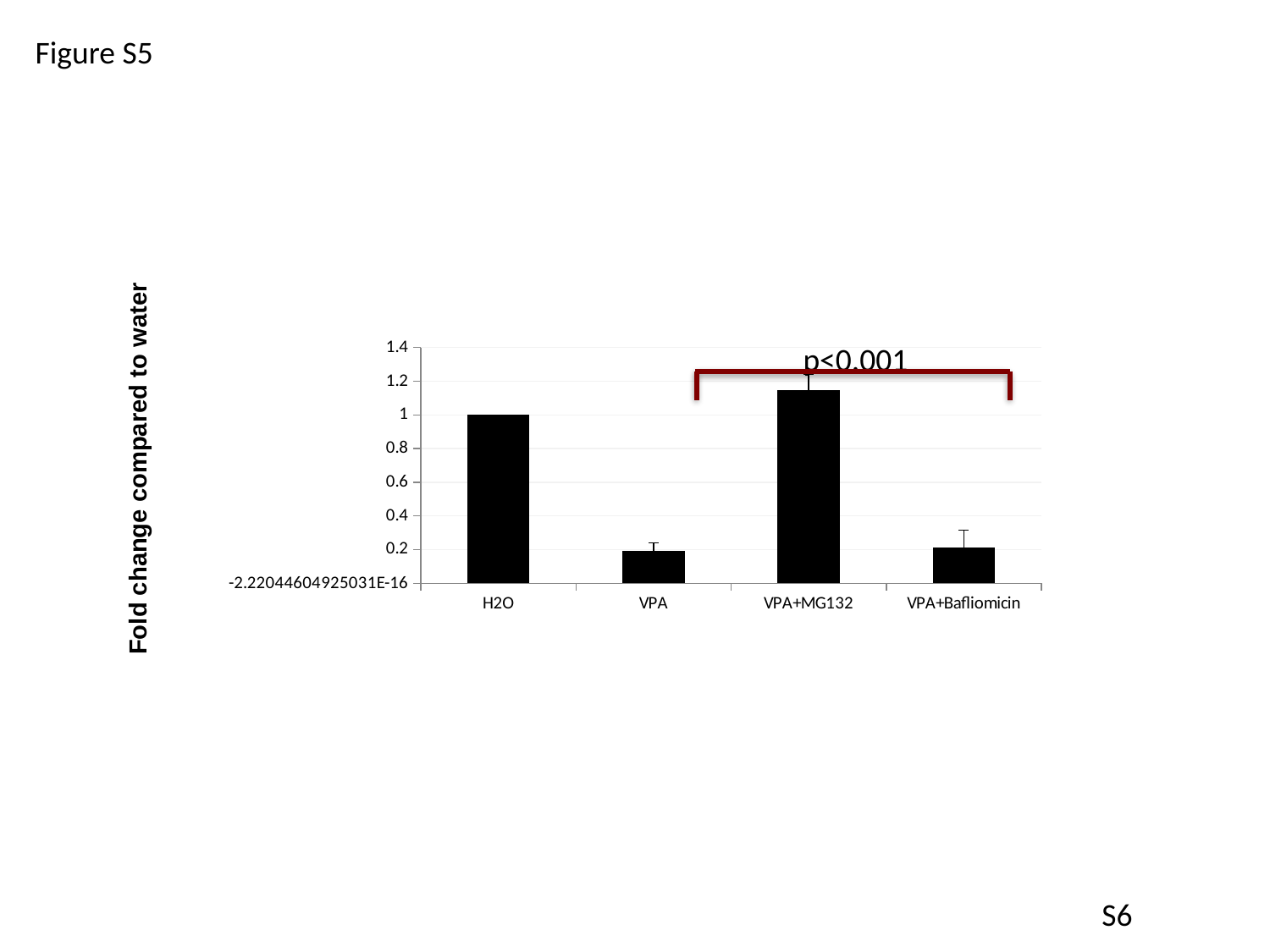

Figure S5
### Chart
| Category | |
|---|---|
| H2O | 1.0 |
| VPA | 0.190312538136866 |
| VPA+MG132 | 1.148711688508892 |
| VPA+Bafliomicin | 0.212160633351339 |p<0.001
Fold change compared to water
S6

Supplement: Figure S5 — Quantification of the effect of MG132 and Bafliomycin on VPA mediated DICER degradation. The DICER signal was normalized to beta-actin and the ratio in the water treated control was set to 1; n=3. A representative experiment is shown in Figure 4B. (PPTX) [file pone.0082895.s005.pptx]
