# Supplementary material for: Valproic Acid Causes Proteasomal Degradation of DICER and Influences miRNA Expression
Source: PLoS One. 2013 Dec 17;8(12):e82895. doi: 10.1371/journal.pone.0082895 (PMC3866160; doi:10.1371/journal.pone.0082895)
Supplement: Figure S6 — Summary of miRNAs changes after VPA treatment. HEK293 cells were treated with 20 mM VPA and changes in miRNAs monitored by array analysis. The table lists miRNAs with the highest fold-changes and their experimentally validated target genes from the literatures. (PPTX) [file pone.0082895.s006.pptx]

## Slide 1
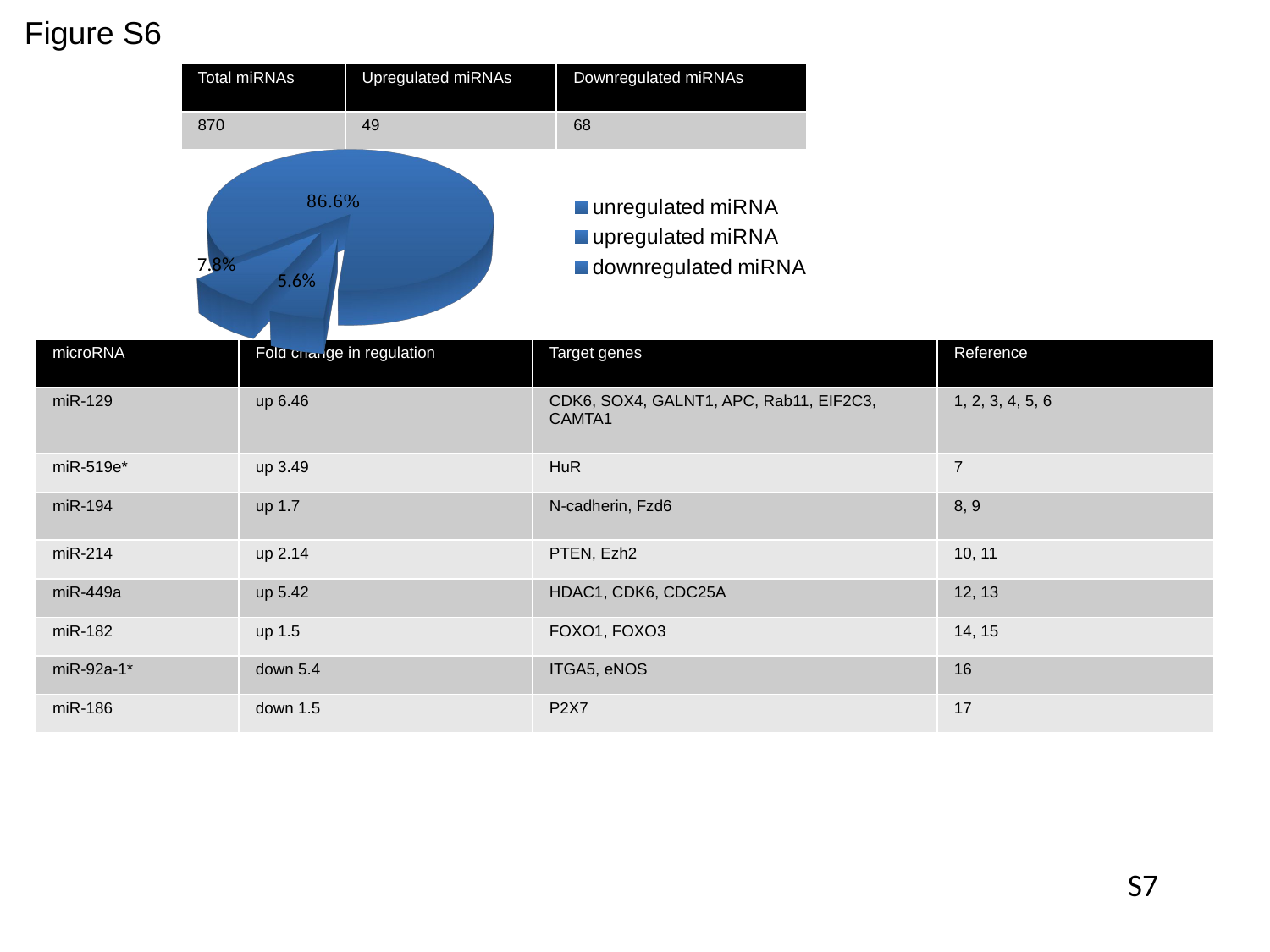

Figure S6
| Total miRNAs | Upregulated miRNAs | Downregulated miRNAs |
| --- | --- | --- |
| 870 | 49 | 68 |
[unsupported chart]
7.8%
5.6%
| microRNA | Fold change in regulation | Target genes | Reference |
| --- | --- | --- | --- |
| miR-129 | up 6.46 | CDK6, SOX4, GALNT1, APC, Rab11, EIF2C3, CAMTA1 | 1, 2, 3, 4, 5, 6 |
| miR-519e\* | up 3.49 | HuR | 7 |
| miR-194 | up 1.7 | N-cadherin, Fzd6 | 8, 9 |
| miR-214 | up 2.14 | PTEN, Ezh2 | 10, 11 |
| miR-449a | up 5.42 | HDAC1, CDK6, CDC25A | 12, 13 |
| miR-182 | up 1.5 | FOXO1, FOXO3 | 14, 15 |
| miR-92a-1\* | down 5.4 | ITGA5, eNOS | 16 |
| miR-186 | down 1.5 | P2X7 | 17 |
S7

## Slide 2
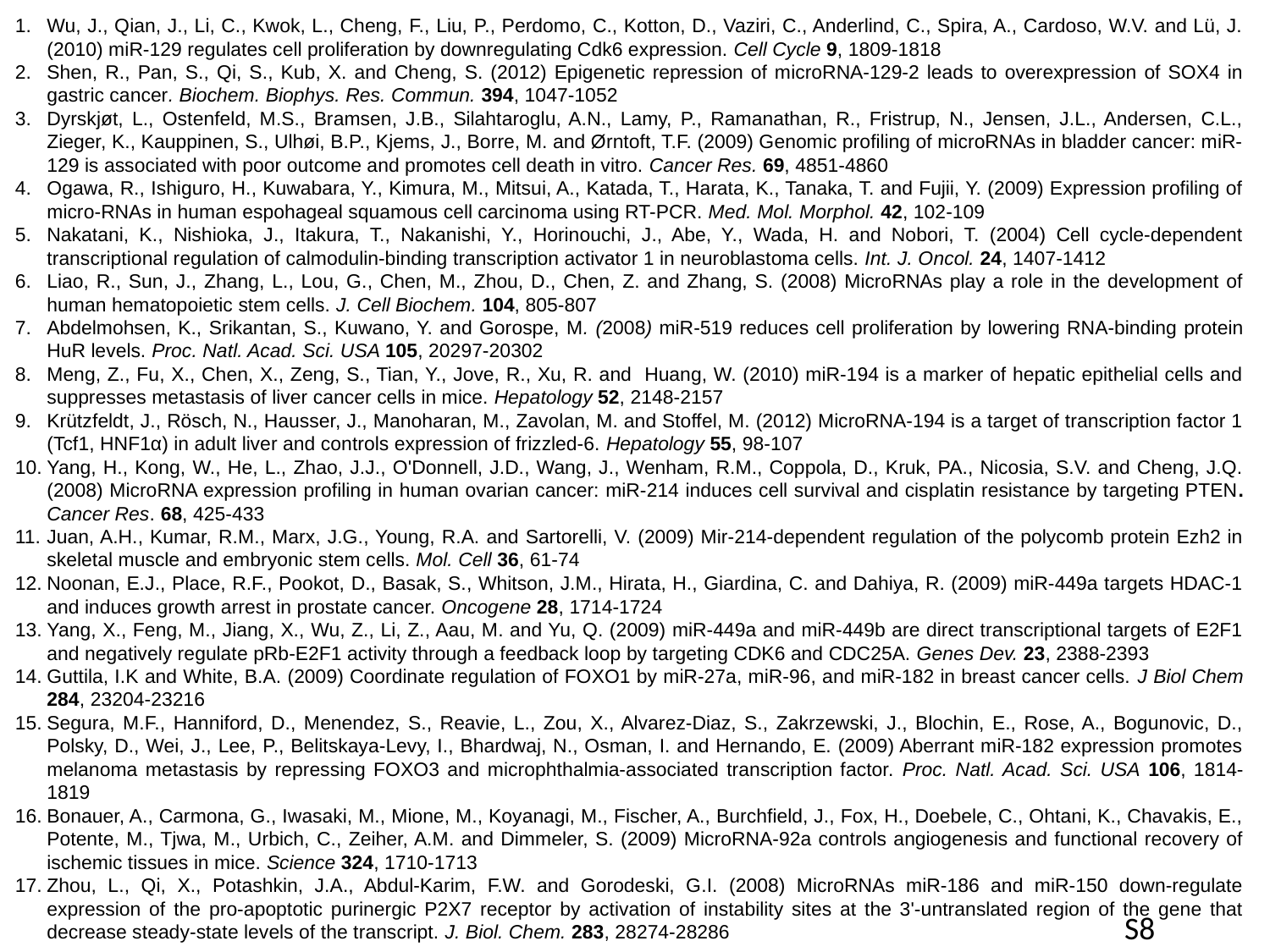

Wu, J., Qian, J., Li, C., Kwok, L., Cheng, F., Liu, P., Perdomo, C., Kotton, D., Vaziri, C., Anderlind, C., Spira, A., Cardoso, W.V. and Lü, J. (2010) miR-129 regulates cell proliferation by downregulating Cdk6 expression. Cell Cycle 9, 1809-1818
Shen, R., Pan, S., Qi, S., Kub, X. and Cheng, S. (2012) Epigenetic repression of microRNA-129-2 leads to overexpression of SOX4 in gastric cancer. Biochem. Biophys. Res. Commun. 394, 1047-1052
Dyrskjøt, L., Ostenfeld, M.S., Bramsen, J.B., Silahtaroglu, A.N., Lamy, P., Ramanathan, R., Fristrup, N., Jensen, J.L., Andersen, C.L., Zieger, K., Kauppinen, S., Ulhøi, B.P., Kjems, J., Borre, M. and Ørntoft, T.F. (2009) Genomic profiling of microRNAs in bladder cancer: miR-129 is associated with poor outcome and promotes cell death in vitro. Cancer Res. 69, 4851-4860
Ogawa, R., Ishiguro, H., Kuwabara, Y., Kimura, M., Mitsui, A., Katada, T., Harata, K., Tanaka, T. and Fujii, Y. (2009) Expression profiling of micro-RNAs in human espohageal squamous cell carcinoma using RT-PCR. Med. Mol. Morphol. 42, 102-109
Nakatani, K., Nishioka, J., Itakura, T., Nakanishi, Y., Horinouchi, J., Abe, Y., Wada, H. and Nobori, T. (2004) Cell cycle-dependent transcriptional regulation of calmodulin-binding transcription activator 1 in neuroblastoma cells. Int. J. Oncol. 24, 1407-1412
Liao, R., Sun, J., Zhang, L., Lou, G., Chen, M., Zhou, D., Chen, Z. and Zhang, S. (2008) MicroRNAs play a role in the development of human hematopoietic stem cells. J. Cell Biochem. 104, 805-807
Abdelmohsen, K., Srikantan, S., Kuwano, Y. and Gorospe, M. (2008) miR-519 reduces cell proliferation by lowering RNA-binding protein HuR levels. Proc. Natl. Acad. Sci. USA 105, 20297-20302
Meng, Z., Fu, X., Chen, X., Zeng, S., Tian, Y., Jove, R., Xu, R. and Huang, W. (2010) miR-194 is a marker of hepatic epithelial cells and suppresses metastasis of liver cancer cells in mice. Hepatology 52, 2148-2157
Krützfeldt, J., Rösch, N., Hausser, J., Manoharan, M., Zavolan, M. and Stoffel, M. (2012) MicroRNA-194 is a target of transcription factor 1 (Tcf1, HNF1α) in adult liver and controls expression of frizzled-6. Hepatology 55, 98-107
Yang, H., Kong, W., He, L., Zhao, J.J., O'Donnell, J.D., Wang, J., Wenham, R.M., Coppola, D., Kruk, PA., Nicosia, S.V. and Cheng, J.Q. (2008) MicroRNA expression profiling in human ovarian cancer: miR-214 induces cell survival and cisplatin resistance by targeting PTEN. Cancer Res. 68, 425-433
Juan, A.H., Kumar, R.M., Marx, J.G., Young, R.A. and Sartorelli, V. (2009) Mir-214-dependent regulation of the polycomb protein Ezh2 in skeletal muscle and embryonic stem cells. Mol. Cell 36, 61-74
Noonan, E.J., Place, R.F., Pookot, D., Basak, S., Whitson, J.M., Hirata, H., Giardina, C. and Dahiya, R. (2009) miR-449a targets HDAC-1 and induces growth arrest in prostate cancer. Oncogene 28, 1714-1724
Yang, X., Feng, M., Jiang, X., Wu, Z., Li, Z., Aau, M. and Yu, Q. (2009) miR-449a and miR-449b are direct transcriptional targets of E2F1 and negatively regulate pRb-E2F1 activity through a feedback loop by targeting CDK6 and CDC25A. Genes Dev. 23, 2388-2393
Guttila, I.K and White, B.A. (2009) Coordinate regulation of FOXO1 by miR-27a, miR-96, and miR-182 in breast cancer cells. J Biol Chem 284, 23204-23216
Segura, M.F., Hanniford, D., Menendez, S., Reavie, L., Zou, X., Alvarez-Diaz, S., Zakrzewski, J., Blochin, E., Rose, A., Bogunovic, D., Polsky, D., Wei, J., Lee, P., Belitskaya-Levy, I., Bhardwaj, N., Osman, I. and Hernando, E. (2009) Aberrant miR-182 expression promotes melanoma metastasis by repressing FOXO3 and microphthalmia-associated transcription factor. Proc. Natl. Acad. Sci. USA 106, 1814-1819
Bonauer, A., Carmona, G., Iwasaki, M., Mione, M., Koyanagi, M., Fischer, A., Burchfield, J., Fox, H., Doebele, C., Ohtani, K., Chavakis, E., Potente, M., Tjwa, M., Urbich, C., Zeiher, A.M. and Dimmeler, S. (2009) MicroRNA-92a controls angiogenesis and functional recovery of ischemic tissues in mice. Science 324, 1710-1713
Zhou, L., Qi, X., Potashkin, J.A., Abdul-Karim, F.W. and Gorodeski, G.I. (2008) MicroRNAs miR-186 and miR-150 down-regulate expression of the pro-apoptotic purinergic P2X7 receptor by activation of instability sites at the 3'-untranslated region of the gene that decrease steady-state levels of the transcript. J. Biol. Chem. 283, 28274-28286
S8
