# Supplementary material for: Valproic Acid Causes Proteasomal Degradation of DICER and Influences miRNA Expression
Source: PLoS One. 2013 Dec 17;8(12):e82895. doi: 10.1371/journal.pone.0082895 (PMC3866160; doi:10.1371/journal.pone.0082895)
Supplement: Figure S8 — Primers used in the Exon-junction array validation by RT-PCR. (PPTX) [file pone.0082895.s008.pptx]

## Slide 1
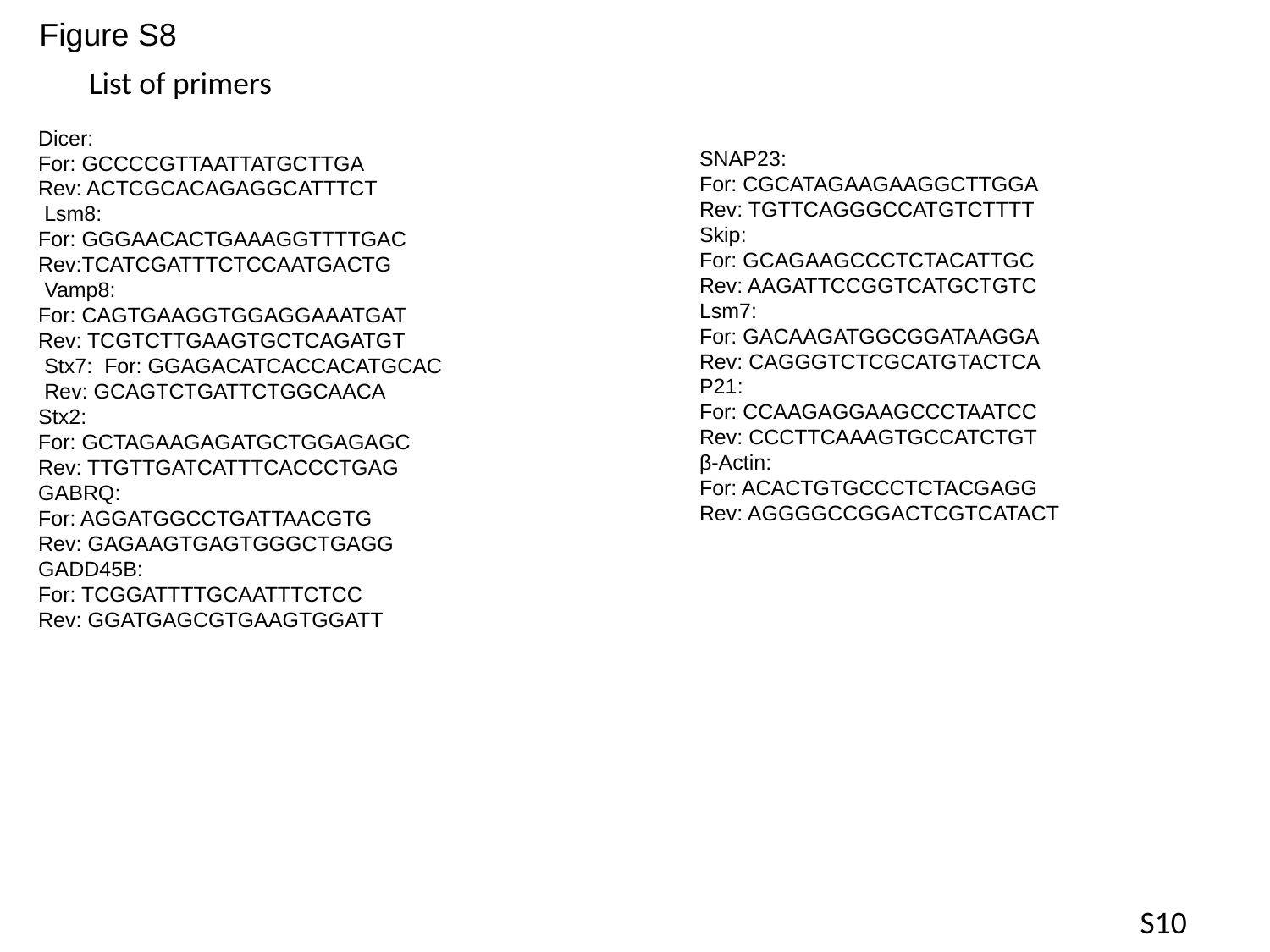

Figure S8
List of primers
Dicer:
For: GCCCCGTTAATTATGCTTGA
Rev: ACTCGCACAGAGGCATTTCT
 Lsm8:
For: GGGAACACTGAAAGGTTTTGAC
Rev:TCATCGATTTCTCCAATGACTG
 Vamp8:
For: CAGTGAAGGTGGAGGAAATGAT
Rev: TCGTCTTGAAGTGCTCAGATGT
 Stx7: For: GGAGACATCACCACATGCAC
 Rev: GCAGTCTGATTCTGGCAACA
Stx2:
For: GCTAGAAGAGATGCTGGAGAGC
Rev: TTGTTGATCATTTCACCCTGAG
GABRQ:
For: AGGATGGCCTGATTAACGTG
Rev: GAGAAGTGAGTGGGCTGAGG
GADD45B:
For: TCGGATTTTGCAATTTCTCC
Rev: GGATGAGCGTGAAGTGGATT
SNAP23:
For: CGCATAGAAGAAGGCTTGGA
Rev: TGTTCAGGGCCATGTCTTTT
Skip:
For: GCAGAAGCCCTCTACATTGC
Rev: AAGATTCCGGTCATGCTGTC
Lsm7:
For: GACAAGATGGCGGATAAGGA
Rev: CAGGGTCTCGCATGTACTCA
P21:
For: CCAAGAGGAAGCCCTAATCC
Rev: CCCTTCAAAGTGCCATCTGT
β-Actin:
For: ACACTGTGCCCTCTACGAGG
Rev: AGGGGCCGGACTCGTCATACT
S10
